# Improving saccharification of ramie stalks by synergistic effect of in-house cellulolytic enzymes consortium

**DOI:** 10.1186/s13568-022-01453-3

**Published:** 2022-09-16

**Authors:** Cha Cao, Zuohua Zhu, Chao Xu, Wenbing Gong, Yingjun Zhou, Li Yan, Zhenxiu Hu, Chunliang Xie, Yuande Peng

**Affiliations:** Institute of Bast Fiber Crops, Chinese Academy of Agricultural Sciences, Changsha, 410205 People’s Republic of China

**Keywords:** Enzyme cocktail, Synergistic effect, Enzymatic hydrolysis, Carbon source, Ramie stalks

## Abstract

The high cost of cellulase is one of the main obstacles hindering the large-scale biorefining of lignocellulosic biomass. Therefore, developing efficient method for preparation of cellulase is promising. In the present study, the production of cellulase by *Trichoderma reesei*, *Trichoderma harzianum*, and *Aspergillus niger* was optimized, and the synergistic effect of these cellulase on enzymatic hydrolysis of pretreated ramie stalks was also evaluated. The maximum CMCase (Carboxymethyl Cellulase) and filter paper activity (FPA) produced by *T. reesei* reached to 3.12 IU/mL and 0.13 IU/mL, respectively. The maximum activities of CMCase (3.68 IU/mL), FPA (0.04 IU/mL) and β-glucosidase (8.44 IU/mL) were obtained from *A. niger*. The results also showed that under the premise of the same FPA activity, the contribution of β-glucosidase activity to yield of reducing sugar was greater than that of CMCase. Besides, cellulase produced by *T. reesei* and *A. niger* had the best synergistic effect on enzymatic hydrolysis of pretreated ramie stalks. The highest reducing sugars yield (417 mg/g dry substrate) was achieved when enzyme cocktail was prepared at the ratio of 1:1, which was 1.36–3.35 folds higher than that of different single enzymes. The present research has provided a novel method for efficient preparation of enzymes consortium for enzymatic hydrolysis of ramie stalks.

**Graphical Abstract:**

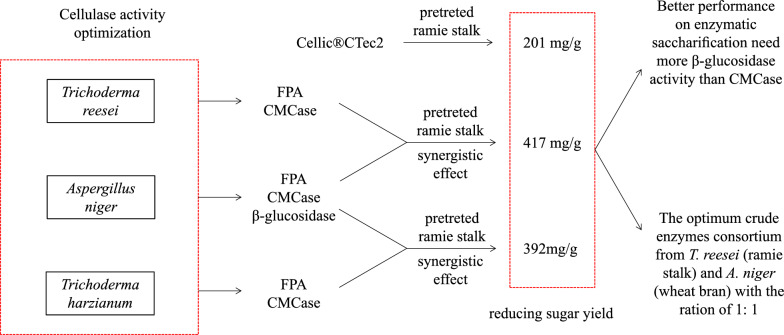

**Supplementary Information:**

The online version contains supplementary material available at 10.1186/s13568-022-01453-3.

## Introduction

With the decrease of fossil fuels reserves and the increase of severe global environmental problems, the use of the renewable lignocellulosic feedstocks as alternative fuel has aroused widespread interest (Mohapatra et al. 2018). The bioconversion of lignocellulosic biomass to target products mainly via three steps: (1) pretreatment; (2) enzymatic hydrolysis; and (3) microbial fermentation (Chen et al. 2021; Raharja et al. 2019). In specific, enzymatic hydrolysis of lignocellulosic biomass to release fermentable sugars is one of the main steps during the process of bio-refinery. As one of the key enzymes for enzymatic hydrolysis of lignocellulose, cellulase is composed of endoglucanase, cellobiohydrolase and β-glucosidase (Okal et al. 2020). During enzymatic digestion process, endoglucanase randomly hydrolyzes the glycosidic bonds in the cellulose chain to provide short chains; cellobiohydrolase releases cellobiose from the non-reducing end of the chain; and the cellobiose further be hydrolyzed by β-glucosidase to produce monosaccharides (Li et al. 2019). However, due to the lack of efficient microorganisms to produce cellulase, the cost for saccharification of lignocellulose remains very high. Therefore, it is essential to explore a cheap and efficient method to prepare cellulase to reduce the cost of biomass refining (Elsa et al. 2015).

Currently, commercial cellulases mainly produced by *Trichoderma* spp. which can only produce limited β-glucosidase, while the strains *Aspergillus* spp. was commonly used for β-glucosidase production(Das et al. 2013; Dias et al. 2018). So far, it is impossible to efficiently produce all kinds of enzymes that meets the requirement of enzymatic hydrolysis of lignocellulose by using single natural microorganism. It is considered a feasible solution to compound the enzymes produced by different strains or to modify the enzyme producing pathways of the strains through genetic engineering (Dionisi et al. 2015). So far, synergistic effect of cellulase consortium was proved to eliminate feedback inhibition and boost enzymatic saccharification (Donohoe and Resch 2015). For example, enzymes extracted from *Penicillium janthinellum* EMS-UV-8, *T. reesei* RUT-C30 and *A. tubingensis* were used to co-hydrolysis avicel-wheat bran. The obtained reducing sugars after hydrolyzing by the three enzymes mixture were two times higher than that of single enzyme at the same enzyme dosages (Adsul et al. 2014). Besides, compared with single enzyme, the mixtures of *P. chrysogenum* P33 and commercial cellulase exhibited synergistic effect on improving the enzymatic hydrolysis of delignified corn stover (Yang et al. 2018).

Ramie (*Boehmeria nivea* L. Gaud) is widely planted in China, India, and other countries of Southeast Asian countries to provide high-quality natural fiber and feed (Wang et al. 2018). It can be planted on marginal land, prevent soil erosion and fix a large amount of greenhouse gases CO_2_. Its above-ground biomass productivity can reach 14–20 Mg/ha dry matter (Wang et al. 2018; Zhu et al. 2016). The phloem of ramie is accounted for only about 15% of its biomass, and the stalks reach to more than 60% (Meng et al. 2018). Ramie stalks composed of 44% cellulose, 31% hemicellulose and 18.1% lignin, making it a suitable substrate for biofuel production (Xie et al. 2021). However, the lignin content of native lignocellulosic biomass is highly resistant to cellulase degradation for cellulose, resulting in low cellulose conversion (Guo et al. 2018). Typical pretreatments, such as weak or strong acid, alkaline oxidative and organosolve, have been required for special instrument, energy losses of holocelluloses, environmental unfriendliness and generation inhibitors to the subsequent enzymatic hydrolysis (Norrrahim et al. 2021). Biological pretreatment is a mild, safe and environmental friendly way to remove lignin from lignocellulose. Specially, some studies have indicated that white-rot fungi are the most promising microorganisms used for biological pretreatment based on their abilities to selectively degradation of lignin. Some typical white-rot fungi, such as *P. ostreatus* and *Ceriporiopsis subvermispora* degrade lignin before degrading cellulose, successfully employed to pretreat rice straw and wheat straw for improving enzyme saccharification (Cianchetta et al. 2014; Taniguchi et al. 2005). Biological pretreatment with *Pleurotus eryngii* is necessary before enzymatic saccharification of ramie stalk (Xie et al. 2021). However, there is still a lack of low-cost enzymes that can be used for enzymatic hydrolysis of ramie stalks.

Specific cellulase mixture is not suitable to all materials due to heterogeneity of lignocellulosic feedstocks in terms of composition and physical properties (Du et al. 2018). Thus, in order to reduce the cost of cellulases to enzymatic hydrolysis of ramie stalks, it is necessary to design efficient compound enzymes according to the characteristics of the raw materials(Du et al. 2020; Xu et al. 2019). Present study was aimed to develop an efficient method to produce “in-house” cellulose by *T. harzianum*, *T. reesei* and *A. niger*. After that, the hydrolysis performance of these enzymes was compared with commercial cellulase. The culture conditions (medium composition, pH, temperature and culture time) of fungi were optimized to obtain highest cellulase activity. In addition, the synergistic effect of enzymes produced by *T. harzianum*, *T. reesei* and *A. niger* on bioconversion efficiency of bio-pretreated ramie stalks were also investigated.

## Materials and methods

### Microorganisms and inoculum

*Trichoderma reesei* (CICC2626) was obtained from the BeNA Culture Collection. *Trichoderma harzianum* (CICC41290) was obtained from the China Center of Industrial Culture Collection. *Aspergillus niger* (ATCC16404) was purchased from the American Type Culture Collection. These strains were cultured on PDA medium at 25 °C for 5 days. After growth on the plate, the spores were washed with 3 mL distilled water to obtain spore suspension for further inoculum. The inoculum was prepared by culturing the fungi under submerged fermentation in 300 mL Erlenmeyer flasks, the seed medium was prepared as following (g/L): glucose 10, peptone 1, Tween-80 0.002, ammonium sulfate 1.4, KH_2_PO_4_ 2, urea 0.3, MgSO_4_.7H_2_O 0.3, CaCl_2_ 0.4, ZnSO_4_ 0.0014, MnSO_4_ 0.0016, FeSO_4_ 0.005, citric acid buffer 500 mL/L (Mallikarjunaiah and Bhide 1983).

### Optimization of substrate for cellulase production by *T. harzianum*, *T. reesei* and *A. niger*

Different carbon sources (ramie, wheat bran or avicel) were studied to evaluate their effects on cellulase production from *A. niger*. The concentration of carbon sources was listed as following: ramie (80 g/L), wheat bran (80 g/L), avicel (80 g/L). The fermentation broth containing ammonium sulfate (4.0 g/L), KH_2_PO_4_ (2.0 g/L), MgSO_4_.7H_2_O (0.3 g/L), tryptone (3.0 g/L) and yeast extract (0.5 g/L).

The carbon source including ramie, wheat bran, lactose or avicel, inorganic and organic nitrogen source including ammonium sulphate, ammonium chloride and tryptone, yeast extract for *T. reesei* and *T. harzianum* with submerged fermentation (SmF) were evaluated respectively. The concentrations were set as follows: ramie (10, 20 and 30 g/L), wheat bran (10, 20 and 30 g/L), lactose (10, 20 and 30 g/L), avicel (10, 20 and 30 g/L), ammonium sulphate (4, 6, 8 and 10 g/L), ammonium chloride (4, 6, 8 and 10 g/L), tryptone (1, 2, 3 and 4 g/L) and yeast extract (1, 2, 3 and 4 g/L). The cultivation of *T. reesei* and *T. harzianum* were carried out in the culture also contains(g/L): KH_2_PO_4_, 2.0; MgSO_4_.7H_2_O, 0.3 and Tween 80, 0.002, MnSO_4_ 0.0016, ZnSO_4_ 0.0014 mg and FeSO_4_ 0.005.

The fungi were cultured under agitation of 180 rpm at 30 °C. The extraction of cellulase was performed at 36 h for *T. reesei* and *T. harzianum*, and at 72 h for *A. niger*. The supernatant was collected by filtering through nylon cloth followed by centrifugation at 10,000 rpm for 10 min. All experiments were performed in triplicate.

### Optimization of pH, temperature and fermentation time for cellulase production by *T. harzianum*, *T. reesei* and *A. niger*

*Trichoderma reesei*, *T. harzianum* and *A. niger* fermentation were performed at different pH value (3.0, 4.0, 5.0, 6.0 and 7.0), temperature (25, 30 and 35 °C) and fermentation time (24, 48, 72 and 96 h). The following assay conditions were identical to those aforementioned.

### Measurement of enzyme activities

Carboxymethyl cellulase (CMCase), filter paper activity (FPA) and β-glucosidase were measured as the description of Liao et al. (2015). All of experiments were performed in 0.05 M sodium acetate buffer (pH 4.8). 3,5-dinitrosalicylic acid (DNS) reagent was used to measure the reducing sugar (Liao et al. 2015).

### Pretreatment of ramie stalk

Air-dried ramie stalks from the Institute of Bast Fiber Crops at the Chinese Academy of Agricultural Sciences were cut into small chips (400–800 μm mesh). Three grams of ramie stalk was added into 7 mL H_2_O, 0.001 g/L Tween 80, 4 mmol/L veratryl alcohol, 0.2 mmol/L Mn^2+^ in 300 mL Erlenmeyer flasks and sterilized at 121 °C for 20 min. *Pleurotus eryngii* was cultured in PDA medium at 28 °C for 7 days. *P. eryngii* pre-cultures were inoculated into the substrates and incubated at 28 °C for 21 days. All of the experiments were carried out in triplicate. The content of cellulose, hemicellulose and lignin in pretreated substrate were 35%, 17% and 11%, respectively (Xie et al. 2021).

### Enzymatic hydrolysis

Enzymatic hydrolysis of pretreated ramie stalks was performed using Cellic^®^CTec2 (Bagsvćrd, Denmark), the crude extracts of *T. reesei*, *T. harzianum* and *A. niger* (cultured at the optimal conditions) as enzymes (enzyme dosage was 30 FPU/g dry biomass). Enzyme cocktails were prepared as described in Table 1. Enzymatic hydrolysis was carried out in 50 mL centrifuge tube for 48 h. Supernatant was collected after centrifuging sample at 10,000 rpm for 5 min to measure the reducing sugar.

**Table 1:** The blends of enzyme cultured in different carbon sources and commercial cellulase

Enzyme codes	Combination mode
1	66.7% M1 + 33.3% M2
2	50.0% M1 + 50.0% M2
3	33.3% M1 + 66.7% M2
4	66.7% M1 + 33.3% M3
5	50.0% M1 + 50.0% M3
6	33.3% M1 + 66.7% M3
7	66.7% M2 + 33.3% M3
8	50.0% M2 + 50.0% M3
9	33.3% M2 + 66.7% M3
10	100%M1
11	100%M2
12	100%M3
13	Cellic^®^CTec2

M1: Extracts of *T. reesei* cultured using ramie as carbon source

M2: Extracts of *T. harzianum* cultured using ramie as carbon source

M3: Extracts of *A. niger* cultured using wheat bran as carbon source

Cellic^®^CTec2: The CTEC2 was diluted by 50-fold, and CMCase, FPA and β-glucosidase activities are 3.45, 0.46 and 1.89 IU/mL, respectively

The conversion rate of reducing sugars (mg/g) was calculated as following:

$${\text{Reducing sugars }}\left( {{{{\text{mg}}} \mathord{\left/ {\vphantom {{{\text{mg}}} {\text{g}}}} \right. \kern-\nulldelimiterspace} {\text{g}}}} \right) = {{{\text{reducing sugars obtained }}\left( {{\text{mg}}} \right)} \mathord{\left/ {\vphantom {{{\text{reducing sugars obtained }}\left( {{\text{mg}}} \right)} {{\text{reducing sugars in raw material }}({\text{g}})}}} \right. \kern-\nulldelimiterspace} {{\text{reducing sugars in raw material }}({\text{g}})}}.$$

### Statistical analysis

All the data in this study were presented as mean ± SD, and *t* test was applied to calculate the significances between different groups (significance levels = 0.05).

## Results

### Optimization of fermentation medium for cellulolytic cellulase production

**Fig. 1:**
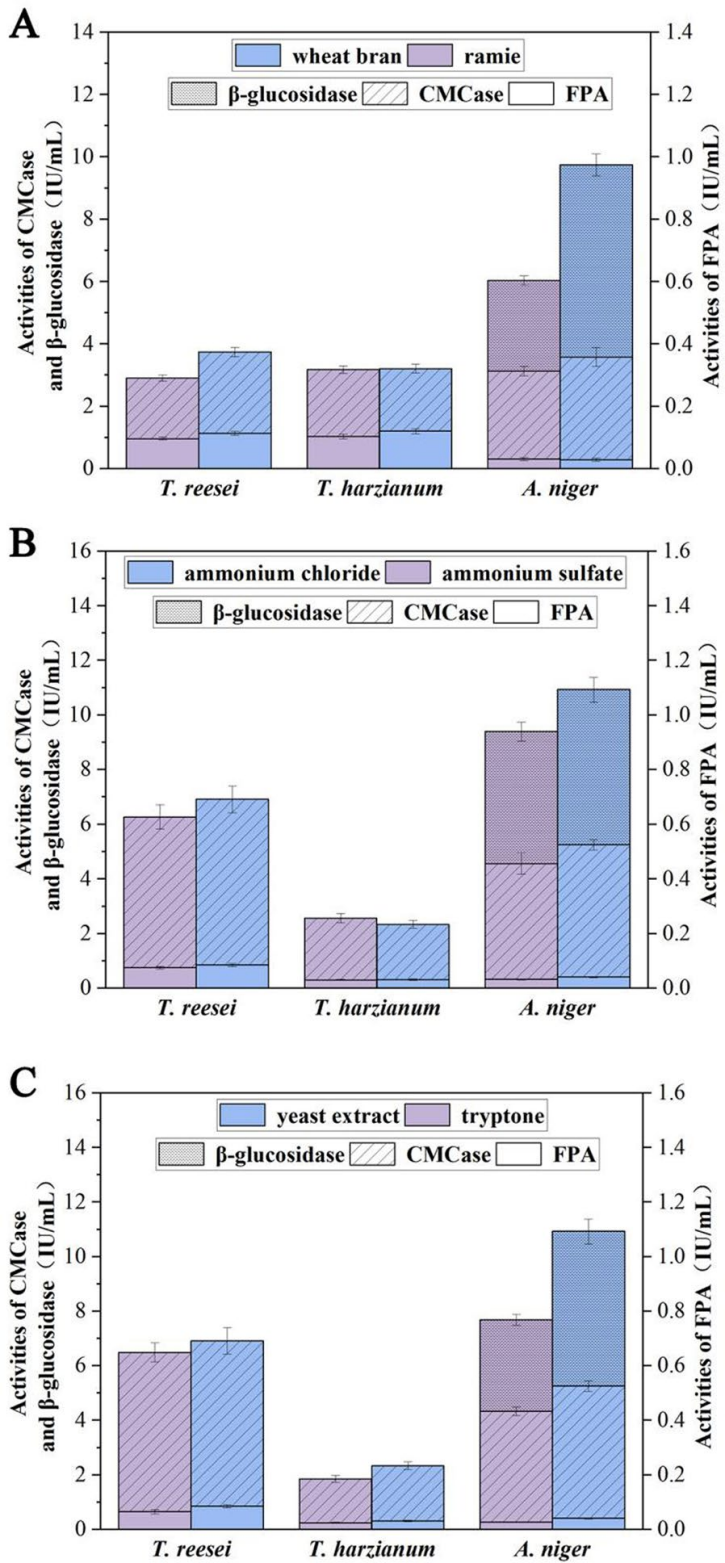
The effects of different sources of carbon (**A**), inorganic (**B**) and organic (**C**) nitrogen on the production of cellulases by different fungi

The optimization of sources of carbon and nitrogen for different fungi to produce cellulases were presented in Fig. 1 and detailed information were as followed in Additional file 1: Figs. S1–S6. The optimal medium components for cellulase production by *T. reesei* contains (g/L): wheat bran 30, ammonium chloride 6 and tryptone 1. The enzyme activity of CMCase and FPA reached 2.61 IU/mL and 0.11 IU/mL, respectively. The medium for *T. harzianum* was optimized as following: ramie 30 g/L, ammonium sulfate 6 g/L and tryptone 1 g/L. At this condition, CMCase and FPA activities can reach 2.14 IU/mL and 0.10 IU/mL respectively. The optimal substrate for *A. niger* to produce cellulase was as following: wheat bran 80 g/L, (NH_4_)_2_SO_4_ 6 g/L and yeast extract 4 g/L. The achieved enzyme activities of FPA, CMCase and β-glucosidase were 0.03 IU/mL, 3.30 IU/mL and 6.16 IU/mL, respectively.

### Effect of temperature, pH and culture time on cellulase production

**Fig. 2:**
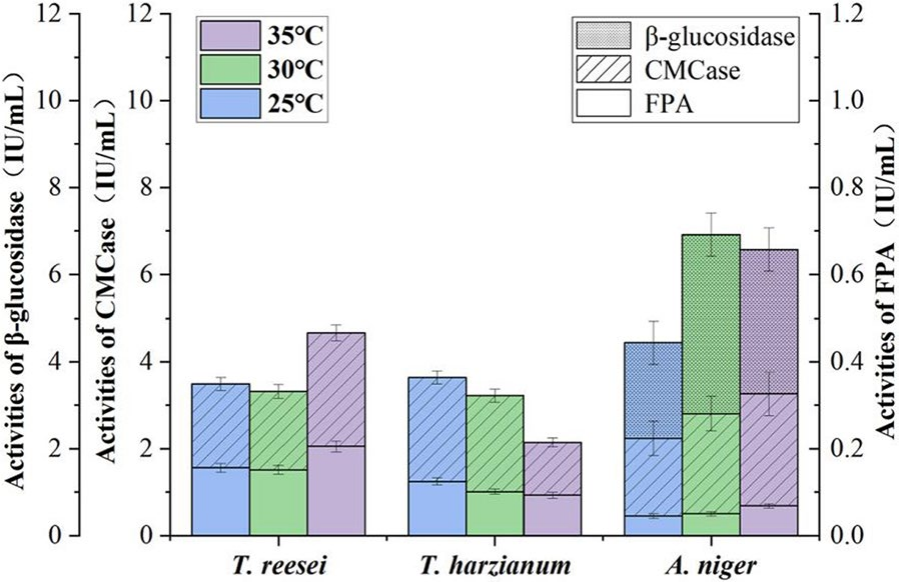
Effects of temperature on the production of cellulases by microorganisms

The effect of temperature on different enzymes production was investigated, which were presented in Fig. 2. When the temperature increased from 25 to 35 °C, the activities of CMCase and FPA from *T. harzianum* were decreased. The highest CMCase and FPA of *T. reesei* was achieved at 35 °C. An enhancement in the activities of CMCase and FPA of *A. niger* were observed as the culture temperature increased from 25 to 35 °C, whereas its maximum β-glucosidase activities (4.11 IU/mL) was obtained at 30 °C.

**Fig. 3:**
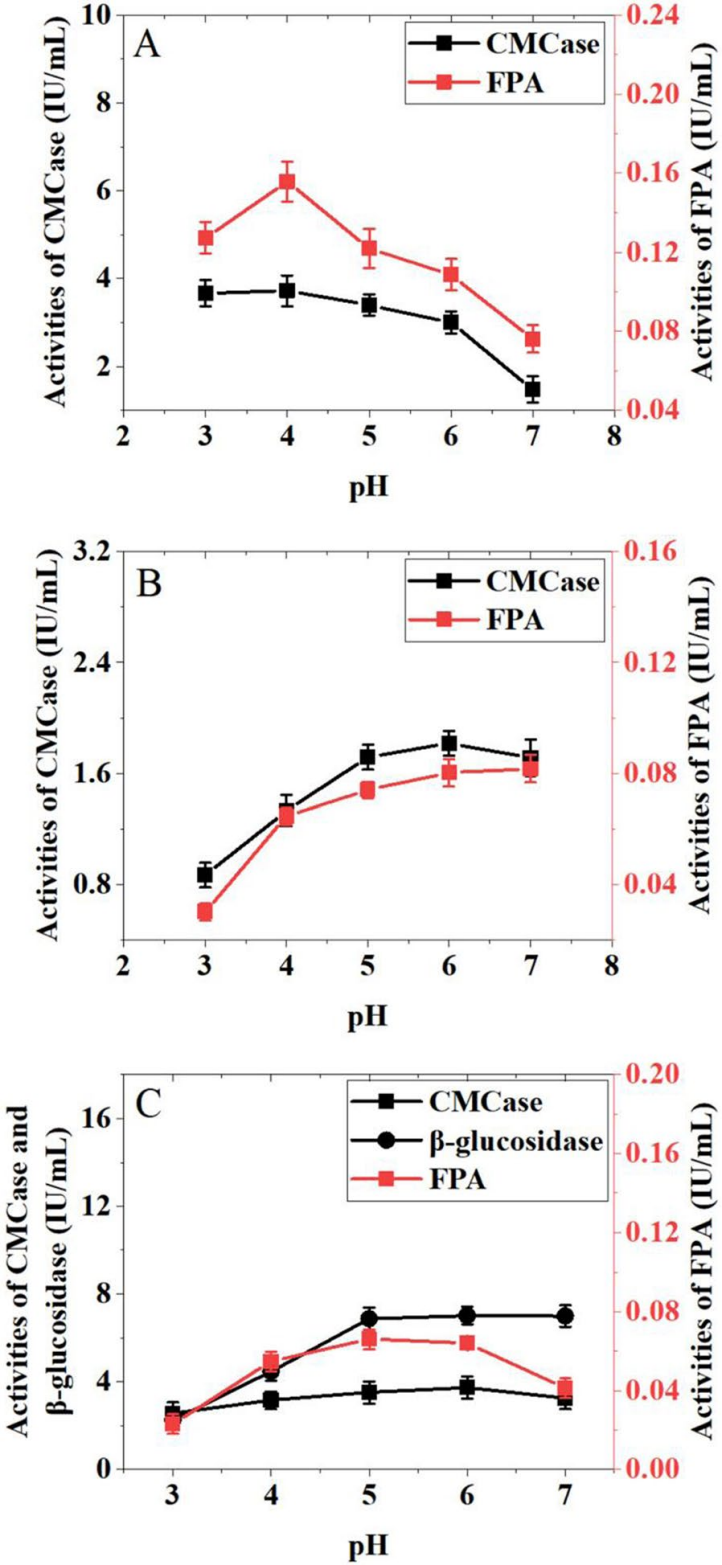
Effects of pH on cellulases production by *T. reesei* (**A**), *T. harzianum* (**B**) and *A. niger* (**C**)

It is well known that initial pH of the medium affects the metabolic ions exchange and the transport characteristic of cell membrane (Diaz et al. 2013). The effect of initial pH value on CMCase, FPA and β-glucosidase producing capability of *T. harzianum*, *A. niger* and *T. reesei* were illustrated in Fig. 3. Results exhibited that both the optimum pH values for CMCase and FPA production by *T. reesei* were at 4.0, while the pH value of 6.0 was more suitable for *A. niger* and *T. harzianum* to produce cellulase.

**Fig. 4:**
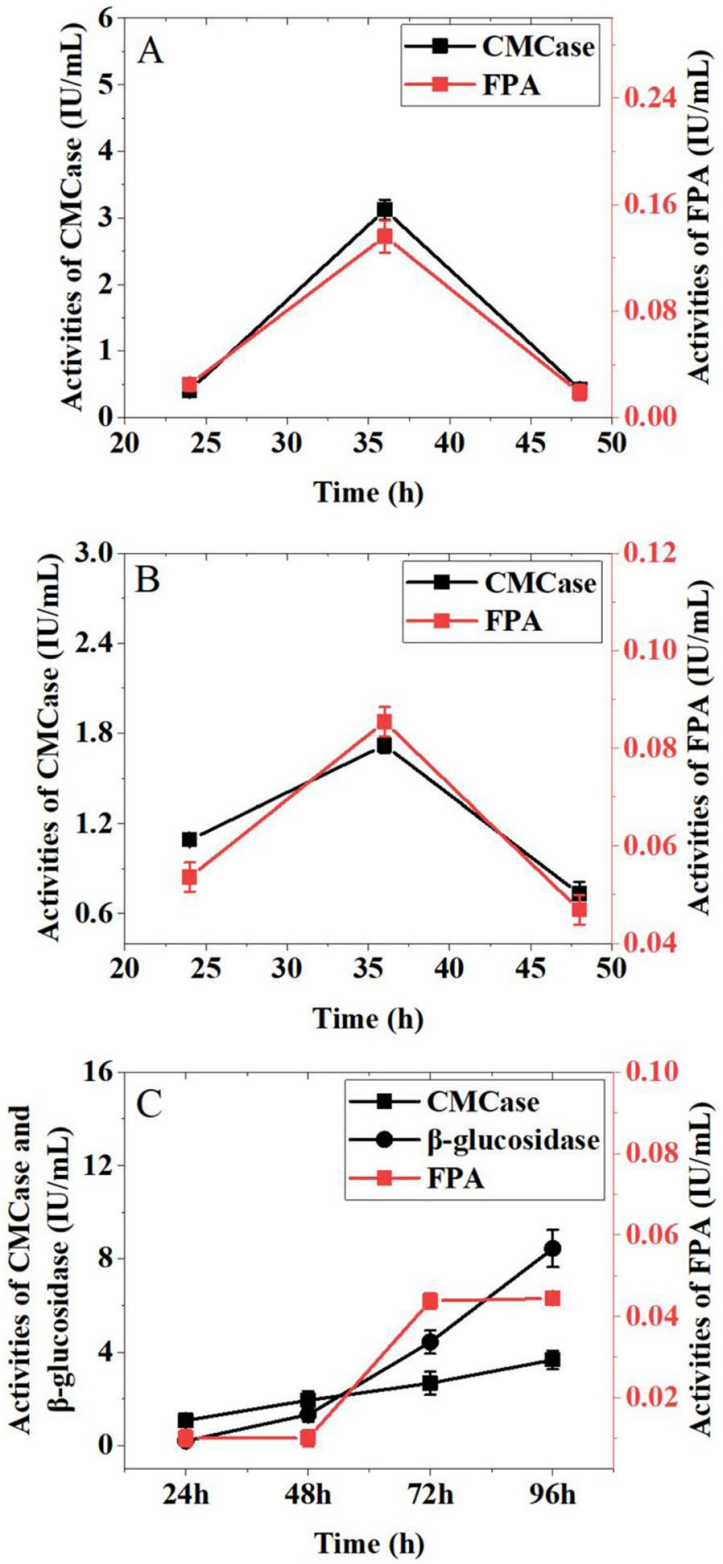
Effects of incubation time on cellulases production by *T. reesei* (**A**), *T. harzianum* (**B**) and *A. niger* (**C**)

In addition, effects of incubation time on production of cellulases were also evaluated (Fig. 4). When the culture time was 36 h, the activities of CMCase (1.72 IU/mL) and FPA (0.08 IU/mL) produced by *T. harzianum* reached the highest. Similarly, the highest activities of CMCase (3.12 IU/mL) and FPA (0.13 IU/mL) produced by *T. reesei* were appeared at 36 h. The maximum activities of CMCase (3.68 IU/mL), FPA (0.04 IU/mL) and β-glucosidase (8.44 IU/mL) were obtained from *A. niger* when it was cultured for 96 h. The final temperature, pH and culture time on cellulase production are shown in Table 2.

**Table 2:** The optimized culture condition(temperature, pH and culture time) of *A. niger*, *T. harzianum* and *T. reesei* and their corresponding enzyme activity(CMCase, FPA and β-glucosidase)

	*Aspergillus niger*	*Trichoderma harzianum*	*Trichoderma reesei*
Culture temperature	30 °C	25 °C	35 °C
Initial pH	6.0	6.0	4.0
Culture time (h)	96	36	36
CMCase (IU/mL)	3.68 ± 0.42	1.72 ± 0.2	3.12 ± 0.52
FPA (IU/mL)	0.04 ± 0.01	0.08 ± 0.02	0.13 ± 0.02
β-glucosidase (IU/mL)	8.44 ± 0.6	0	0

### The influence of cellulases induced by different substrates on enzymatic saccharification of pretreated ramie stalks

**Fig. 5:**
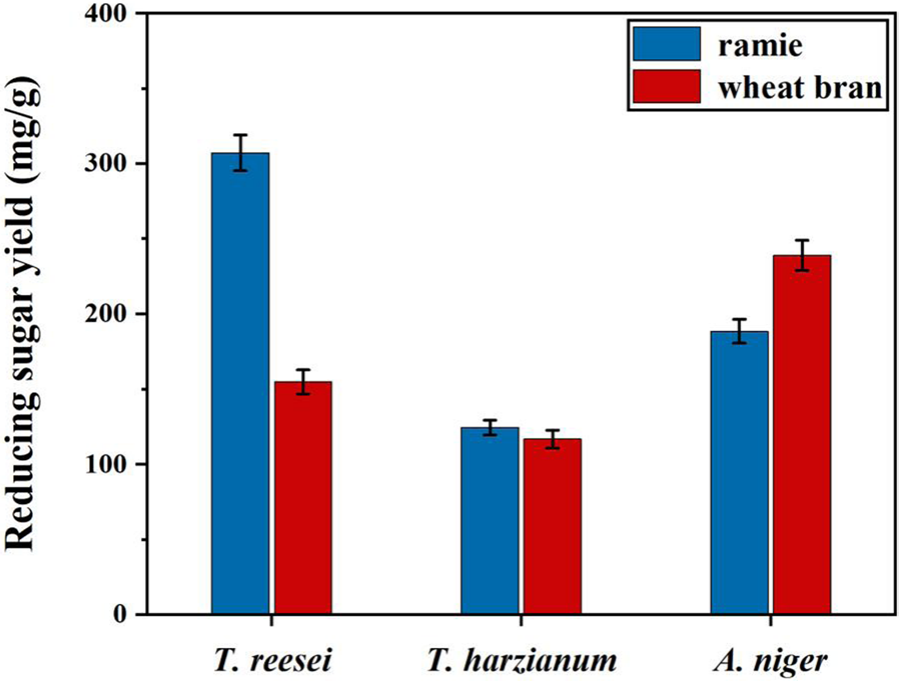
The effect of wheat bran or ramie substrate on the enzymatic hydrolysis efficiency of pretreated ramie stalk with crude enzymes from different microorganisms

As shown in Fig. 1, clear difference in enzyme activities of CMCase, FPA and β-glucosidase by *T. harzianum, A. niger* and *T. reesei* was observed when they were cultured in different lignocellulose substrates. Only with judgement of cellulase activities, it is difficult to choose ramie stalk or wheat bran as candidate inducer for cellulase production, which further applied to enzymatic hydrolysis for pretreated ramie stalk. In our study, cellulases produced by three fungi in wheat bran or ramie substrate were collected, and their effects on enzymatic hydrolysis of pretreated ramie stalk were investigated. Figure 5 showed that the crude enzymes, produced by using ramie stalk to culture *T. reesei,* was applied to enzymatic hydrolysis of the pretreated ramie stalk for 48 h, a reducing sugar yield of 307 mg/g was obtained which was 1.98 times higher than that of wheat bran. However, a reverse result was observed in *A. niger*. Surprisingly, there was no significant difference in reducing sugars yield which was obtained after enzymatic hydrolysis the pretreated ramie stalk using the crude enzymes of *T. harzianum* induced by wheat bran or ramie. Combining of results from Figs. 1 and 5, it was showed that under the premise of the same FPA activity, the contribution of β-glucosidase activity to yield of reducing sugar was greater than that of CMCase.

### Synergistic effect of cellulases consortium

**Fig. 6:**
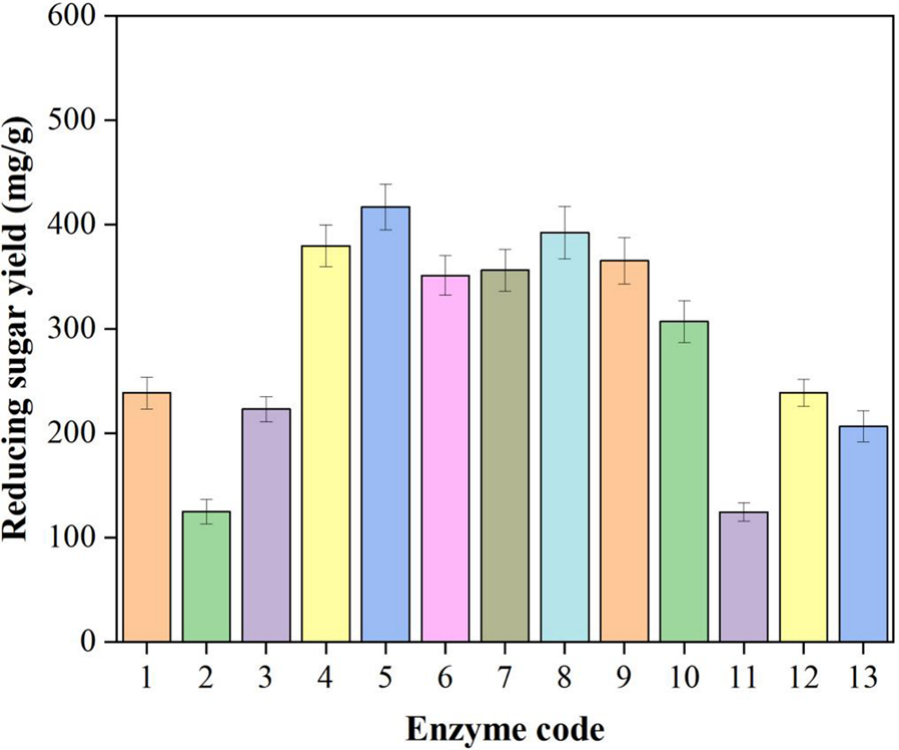
Enzymatic hydrolysis of pretreated ramie stalks by different cellulase consortiums (The number of the horizontal coordinates corresponding to enzyme code of Table 1)

Synergistic effects between enzymes help to reduce loading of cellulase during enzymatic hydrolysis of lignocellulosic biomass. In the present study, the synergistic effects of crude enzyme preparations from *T. harzianum*, *A. niger* and *T. reesei* were evaluated. Figure 6 showed that the highest reducing sugar yield was achieved by enzyme cocktails of No. 5 (50% extracts of *T. reesei* cultured using ramie as carbon source + 50% extracts of *A. niger* cultured using wheat bran as carbon source). The intermediate reducing sugar was obtained when it was digested by No. 8 (50% extracts of *T. harzianum* cultured using ramie as carbon source + 50% extracts of *A. niger* cultured using wheat bran as carbon source), while only very limited reducing sugar was obtained when the substrate was hydrolyzed by the enzyme cocktail of No. 2 (50% extracts of *T. reesei* cultured using ramie as carbon source + 50% extracts of *T. harzianum* cultured using ramie as carbon source). These results suggesting that the synergistic effect on enzyme system of *T. harzianum*, *A. niger* and *T. reesei* can increase the releasing of reducing sugar from pretreated ramie stalks.

## Discussion

The production of cellulosic ethanol depends on the conversion rate of lignocellulose to fermentable sugars through enzymatic hydrolysis (Houfani et al. 2020). Several commercial cellullase (Accelerase, Celluclast 1.5L, CellicCTec2, Novozyme 188) can be used for the saccharification of lignocellulose. Although the use of commercial enzymes to obtain fermentable sugars from lignocellulose has achieved ideal results, the high price is a limiting factor for its large-scale application (Peciulyte et al. 2017; Xue et al. 2015). Besides, a specific enzyme mixture does not show the best performance for every type of biomass because of the diversity of compositional variability (Sharma et al. 2015). Therefore, it is of great significance to develop an enzyme or enzyme mixture with low-cost and good performance for enzymatic hydrolysis of ramie stalks.

Filamentous fungi, especially *Aspergillus* and *Trichoderma *spp., are commonly used for the production of cellulase due to their high enzyme yield, good safety and strong production capacity (Maitan-Alfenas et al. 2015; Xia et al. 2018). In present research, crude enzymes consortium was produced by cultivating *A. niger, T. harzianum* and *T. reesei* in different carbon sources including ramie stalks and wheat bran. An ideal carbon source for these three fungi should both provide the energy required for growth and also induce the efficient expression of the cellulase. For example, Previous studies also used wheat bran, lactose, avicel or other lignocellulose materials (such as leaves, orange peelings, pineapple peelings and sugarcane bagasse) to induce cellulase. Wheat bran appeared to be the best suitable substrate for *A. niger*, *T. reesei* or *T. harzianum*. Wheat bran was used as carbon substrate for *A. niger* NS-2 and its appreciable yields of CMCase, FPA and β-glucosidase reached 310, 17 and 33 U/g dry substrate respectively (Bansal et al. 2012). Wang et al. (2021) systematically analyzed the major secretome components of *T. reesei* RUT-C30 under wheat bran solid-state fermentation and found that wheat bran induced more β-1,4-glucosidases than rice bran. Wheat bran was used as carbon source for solid fermentation of *T. harzianum* (Zhang and Yang 2015). Avicel was also used for cellulase production by *A. niger*. However, the levels of endoglucanase and exoglucanase produced by cultivating *A. niger* in avicel were low. In another study, wheat bran was used for cellulase production by *T. reesei* QM9414. Using 4% untreated wheat bran, this strain produces about 0.50 IU/mL FPase and 2.19 mg/mL protein (Cui et al. 2014). Our results showed that wheat bran was the most beneficial carbon sources to induce cellulase production by *A. niger* and *T. reesei*. However, previous studies only focused on cellulase production by different microorganisms and monitored their enzyme activity, but did not characterize the enzymatic saccharification capacity of lignocellulosic biomass, which may provide important information for its industrial application potential (Boakye-Boaten et al. 2017; Cannella and Jorgensen 2014; Geng et al. 2015; Koppram et al. 2014).

Development of efficient cellulase cocktail would favor to reduce the enzymes dosage and enhance the conversion rate of lignocellulosic feedstock (Adsul et al. 2014). The combination of enzymes produced by *A. niger* and *T. reesei* was proved to boost enzymatic hydrolysis of cellulose substrates(van den Brink et al. 2014). A previous study showed that *T. reesei* was an ideal cellulase producing candidate, unfortunately, although a high total cellulase activity (1.7 FPU/mL) could be obtained using dairy manure as substrate, only very limited β-glucosidase was monitored (Wen et al. 2005). High β-glucosidase production could be obtained by *A. niger* using pretreated wheat straw (WS) and sugarcane bagasse (SCB) as substrates (van den Brink et al. 2014). Similar to previous reports, synergistic effect was observed between the cellulase produced from *T. reesei* and *A. niger* in present research. It is worth mentioning that the optimized enzyme consortiums have exhibited comparable performance with Cellic^®^CTec2 on enzymatic hydrolysis of pretreated ramie stalks.

In addition to enzyme activity, many works have shown that enzyme composition and ration also played an important role on synergistic actions. Oscar Garcia-Kirchner analyzed the saccharification progress of the native sugar cane bagasse pith, and the results indicated a synergistic effect for the mixture of all the enzymes, the highest being in the 40:60 relation of *Penicillium* sp. and *Aspergillus terreus*, respectively (GarciaKirchner and Huitron 1996). It is reported that a blend ratio of 1:1.6:1.4 of endoglucanase: exoglucanase: β-glucosidase was identified as the sweet spot for optimum sugar yield (Obeng et al. 2018). Similary, Fig. 6 showed that the reducing sugar yield obtained with enzyme cocktails combined with *T. reesei* and *A. niger* with a blend ratio of 1:1 was higher than that of non-combinatorial enzyme*.* Moreover, reducing sugar yield from No. 5 was higher than that of No. 6 or No. 4, suggesting that enzyme mixture ratios had a more significant influence in final reducing sugar yield. More importantly, no previous study has used the enzyme cocktails combined with *T. harzianum* and *A. niger* to enzymatic saccharification of lignocellulose. Present research showed the enzyme of *T. harzianum* (No. 11) had relatively inefficiencies on enzymatic saccharification (Fig. 6). However, when combined with cellulase from *A. niger*, the remarkable increase on reducing sugar yield was appeared, even close to the No. 5. It was suggesting that the contribution of β-glucosidase activity to yield of reducing sugar was greater than that of CMCase and FPA. As expected, the combination of *T. reesei* and *T. harzianum* performed the poorest. In addition, after combined analyzed the data from Figs. 1 and 5, we found that there was no causal correlation between the activity of CMCase or FPA and the yield of reducing sugar, while the performance on enzymatic saccharification was positively associated with the level of β-glucosidase in *A. niger*.

In summary, the components of cellulases produced by microorganisms were various when induced by different carbon sources. The cellulases produced by using ramie stalk to induce *T. reesei* had ideal performance on enzymatic saccharification, while wheat bran was more suitable for *A. niger*. Cellulases produced from *T. harzianum*, *A. niger* and *T. reesei* have exhibited obvious synergistic effect on enzymatic hydrolysis of pretreated ramie stalks. The highest reducing sugars was achieved using the crude enzymes produced by *A. niger* and *T. reesei* with a ratio of 1:1, and the reducing sugars concentration was enhanced by 1.36–3.35 folds as compared with different single enzymes. Our research provided a cheap and efficient method for the preparation of cellulases, which is of great significance to the large-scale bio-refining of lignocellulosic biomass.

## Supplementary Information

**Additional file 1: Figure S1.** Effect of different carbon sources on enzyme production by cultivating *T. reesei*. **Figure S2.** Effect of different carbon sources on enzyme production by cultivating *T. harzianum.* **Figure S3.** Effect of different carbon sources on enzyme production by cultivating *A. niger.* **Figure S4.** Effect of different nitrogen sources on enzyme production by cultivating *T. reesei.* **Figure S5.** Effect of different nitrogen sources on enzyme production by cultivating *T. harzianum.* **Figure S6.** Effect of different nitrogen sources on enzyme production by cultivating *A. niger.*

## Data Availability

All data generated or analysed during this study are included in this published article.
