# Supplementary material for: Improving saccharification of ramie stalks by synergistic effect of in-house cellulolytic enzymes consortium
Source: AMB Express. 2022 Sep 16;12:119. doi: 10.1186/s13568-022-01453-3 (PMC9481857; doi:10.1186/s13568-022-01453-3)
Supplement: Supplementary file 1 — Additional file 1: Figure S1. Effect of different carbon sources on enzyme production by cultivating T. reesei. Figure S2. Effect of different carbon sources on enzyme production by cultivating T. harzianum. Figure S3. Effect of different carbon sources on enzyme production by cultivating A. niger. Figure S4. Effect of different nitrogen sources on enzyme production by cultivating T. reesei. Figure S5. Effect of different nitrogen sources on enzyme production by cultivating T. harzianum. Figure S6. Effect of different nitrogen sources on enzyme production by cultivating A. niger. [file 13568_2022_1453_MOESM1_ESM.pdf]

AMB express

Additional file 1：

Improving saccharification of pretreated ramie stalks by synergistic effect of cellulolytic enzymes consortium from *Aspergillus niger* and *Trichoderma reesei*

Chunliang Xie[*], Cha Cao[*], Chao Xu, Wenbing Gong, Zuohua Zhu, Yingjun Zhou, Li Yan,

Zhenxiu Huand Yuande Peng[**]

Institute of Bast Fiber Crops, Chinese Academy of Agricultural Sciences, Changsha 410205, P.R.China

[*]**These authors contributed equally to this work.**

[**]**Corresponding author:**

Yuande Peng

Institute of Bast Fiber Crops, Chinese Academy of Agricultural Sciences, Changsha 410205, P.R.China

E-mail: ibfcpyd313@126.com

Tel.: +86-731-88998523, Fax: +86-731-88998523

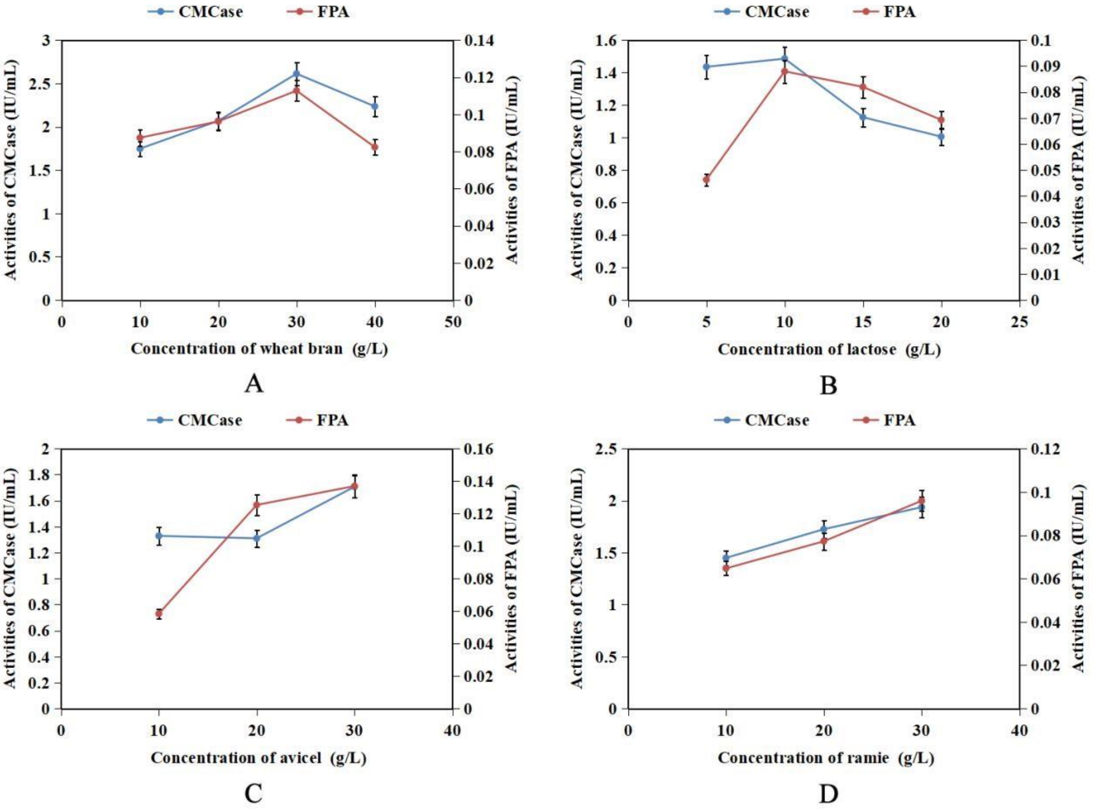

Fig. S1 Effect of different carbon sources on enzyme production by cultivating *T. reesei*.

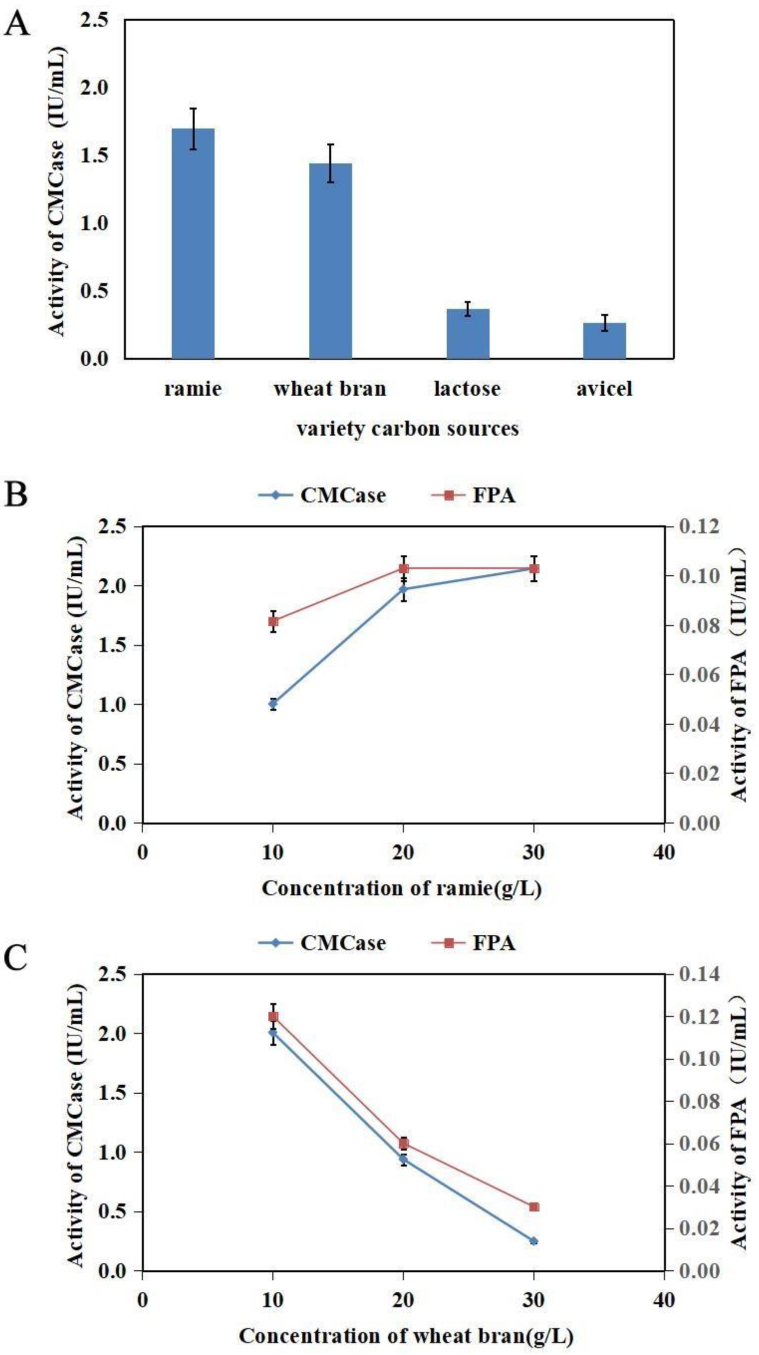

Fig. S2 Effect of different carbon sources on enzyme production by cultivating *T. harzianum.*

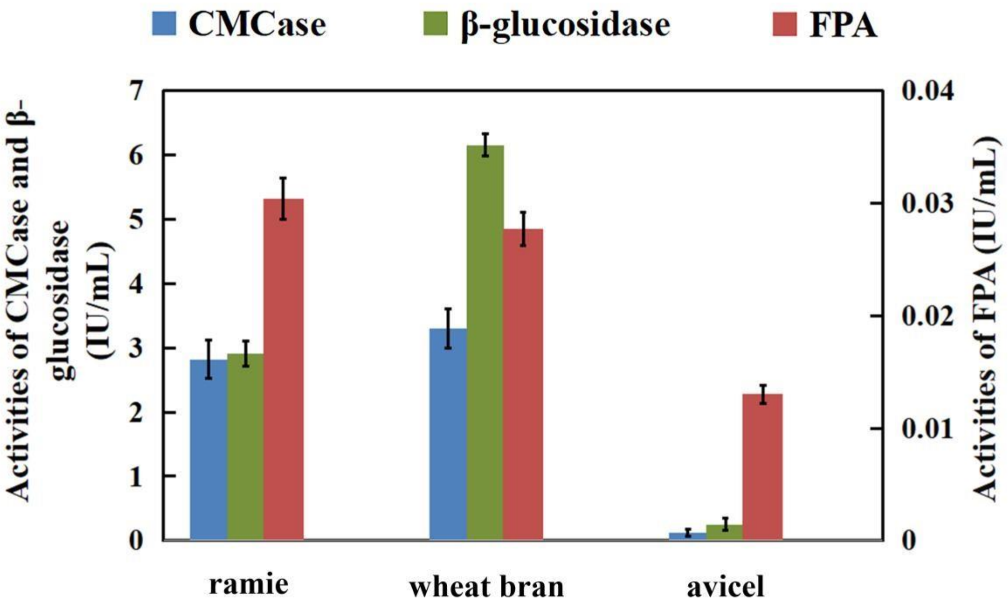

Fig. S3 Effect of different carbon sources on enzyme production by cultivating *A. niger*.

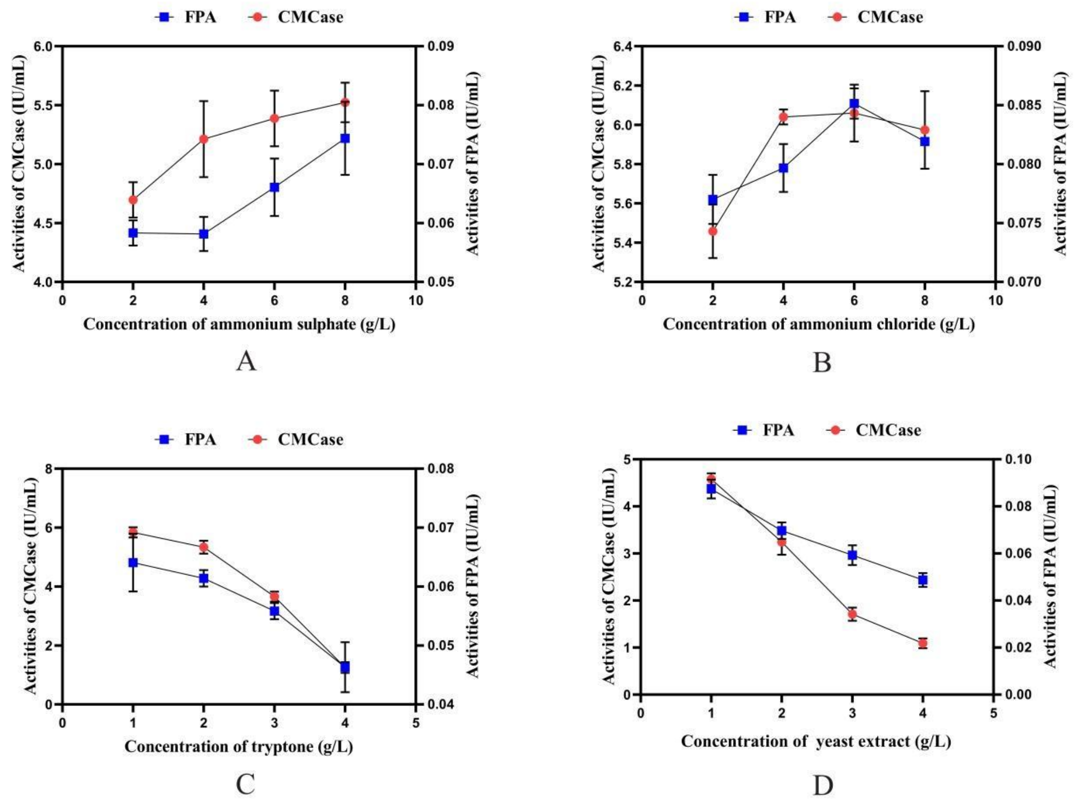

Fig. S4 Effect of different nitrogen sources on enzyme production by cultivating *T. reesei*.

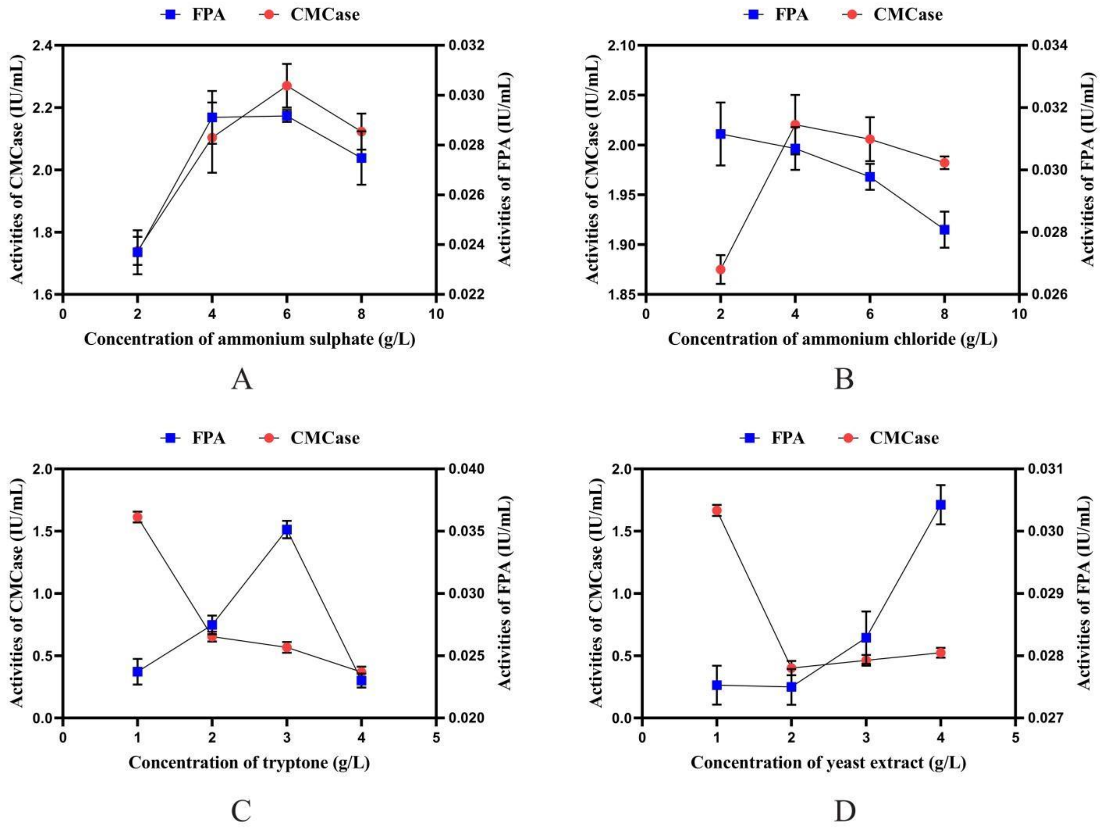

Fig. S5 Effect of different nitrogen sources on enzyme production by cultivating *T. harzianum.*

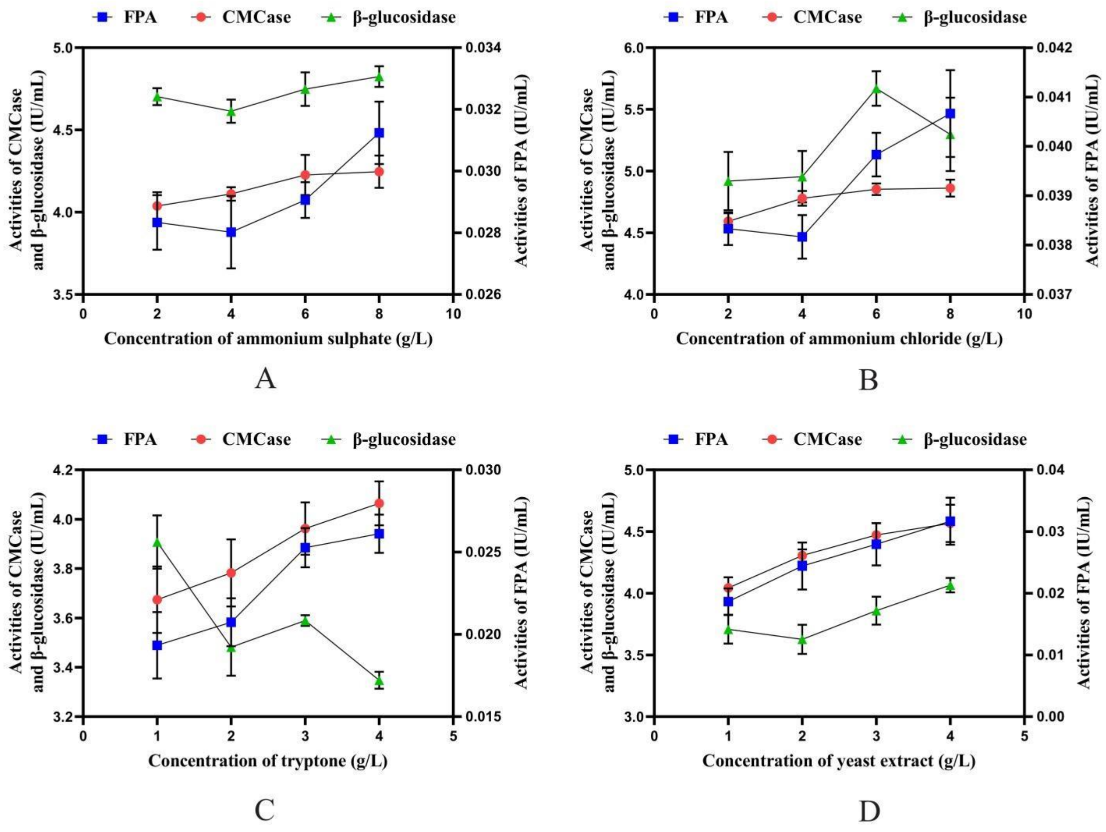

Fig. S6 Effect of different nitrogen sources on enzyme production by cultivating *A. niger*.
